# Parálisis facial aislada posterior a infección por virus de chikunguña: un nuevo diagnóstico diferencial

**DOI:** 10.7705/biomedica.6308

**Published:** 2022-09-02

**Authors:** Augusto Peñaranda, Daniel Peñaranda, María M. Gantiva-Navarro, Lucía C. Pérez-Herrera

**Affiliations:** 1 Departamento de Otorrinolaringología, Fundación Santa Fe de Bogotá, Bogotá, D.C., Colombia Fundación Santa Fe de Bogotá Bogotá D.C Colombia; 2 Facultad de Medicina, Universidad de Los Andes, Bogotá, D.C., Colombia Universidad de los Andes Universidad de Los Andes Bogotá D.C Colombia; 3 Grupo de investigación Otorrino y Audiología, Unidad Médico Quirúrgica de Otorrinolaringología, Bogotá, D.C., Colombia Unidad Médico Quirúrgica de Otorrinolaringología Bogotá D.C Colombia; 4 Departamento de Otorrinolaringología, Fundación Universitaria de Ciencias de la Salud-Hospital de San José, Bogotá, D.C., Colombia Fundación Universitaria de Ciencias de la Salud-Hospital de San José Bogotá D.C. Colombia

**Keywords:** virus de chikunguña, parálisis facial, ecosistema tropical, Chikungunya virus, facial palsy, tropical ecosystem

## Abstract

En las últimas décadas, se ha incrementado el reporte de manifestaciones neurológicas asociadas con la infección por el virus de chikunguña. Se informa el caso de un adulto joven previamente sano que presentó parálisis facial izquierda aislada después de una infección reciente por el virus de chikunguña en el trópico colombiano. Se describen aspectos importantes de la fisiopatología del virus y su tropismo por el sistema nervioso central y periférico, y se sugiere considerar este virus en el diagnóstico diferencial de la parálisis facial en pacientes con infección confirmada por el virus de chikunguña en regiones tropicales endémicas o en aquellos con antecedente de viajes recientes a dichas regiones.

## Abstract (en)

A significant raise in the reports of neurological manifestations due to Chikungunya virus has been described worldwide. Here, we describe a case report of a previously healthy young adult who developed isolated left facial palsy after a confirmed Chikungunya virus infection in the Colombian tropics. We suggest considering this virus as a differential diagnosis for facial palsy in patients with confirmed Chikungunya virus infection who live in endemic regions or report a history of recent travel to these regions.

El virus de chikunguña (CHIKV) es un virus ARN del género *Alphavirus* de la familia Togaviridae, aislado aproximadamente en 1955 ^1^, cuya virulencia y patogenia se han reevaluado a raíz de los brotes recientes en Europa y su expansión global. En Colombia, el virus fue epidémico en el 2015 y, según la Organización Mundial de la Salud (OMS), en el 2017 se reportaron 1.052 casos sospechosos ^1^.

Además de las manifestaciones clínicas típicas de fiebre, mialgias, polialtralgias y cefalea, el virus se ha asociado con manifestaciones neurológicas. Entre las complicaciones neurológicas que involucran el sistema nervioso periférico y el central, las más comunes son el síndrome de Guillain-Barré y la meningoencefalitis ^2^^,^^3^. Este es el primer reporte de parálisis facial izquierda de aparición súbita posterior a una infección confirmada serológicamente por el virus de chikunguña adquirido en el trópico colombiano.

## Presentación de caso

En noviembre del 2017, un hombre de 25 años previamente sano se presentó a consulta de otorrinolaringología con síntomas de debilidad y parestesias en la región hemifacial izquierda.

El paciente trabajaba como médico rural en la ciudad de Cartagena y sus alrededores, y se había levantado esa mañana con la imposibilidad de cerrar el ojo izquierdo o fruncir los labios; además, presentaba hipostesia en el lado izquierdo de su cara. Negó haber presentado síntomas como otalgia, plenitud aural, vértigo y debilidad o parestesias en sus extremidades. No tenía antecedentes médicos relevantes ni cirugías previas, pero refirió un episodio de cinco días de fiebre (39 °C), polialtralgias (predominantemente en rodillas y hombros), mialgias y cefalea, diez días antes del inicio de la parálisis facial. En esa ocasión, fue atendido en el departamento de emergencias donde se le hicieron exámenes paraclínicos completos en sangre, incluida la prueba serológica para el virus del dengue (Duo Dengue Ag-IgG/IgM “ad-bio” Rapid Test™). El hemograma reveló leucocitosis leve (10.900 células/mm^3^) e IgM negativa para dengue.

**Figura 1:**
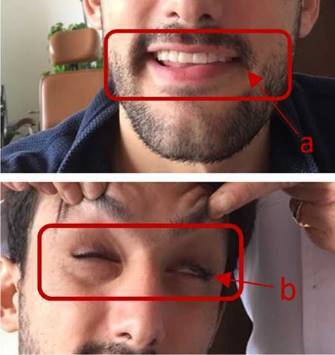
Examen del nervio facial que denota parálisis facial izquierda con clasificación de tipo II en la escala de House-Brackmann: a. Desviación de la comisura labial izquierda. b. Imposibilidad de cerrar el ojo izquierdo completamente

En el examen físico, el paciente parecía tener un buen estado general, pero mostraba incapacidad para cerrar sus ojos o completar movimientos labiales como soplar o inflar sus mejillas (figura 1). Su evaluación otológica, neurológica y oftalmológica fue normal, así como la audiometría, la timpanometría y los reflejos estapediales. En el Servicio de Otorrinolaringología, se hizo un diagnóstico de parálisis facial calificada con 2 según la clasificación de House-Brackmann.

Debido a su historia de trabajo en una ciudad previamente endémica para infecciones por arbovirus, se solicitaron pruebas para detectar los virus del dengue (DENV), de chikunguña (CHIKV) y del Zika (ZIKV). Los títulos de IgM para convalecencia de dengue y la RT-PCR para infección por ZIKV fueron negativos, pero las pruebas para CHIKV fueron positivas, lo cual indicaba que el episodio febril de 10 días habría sido causado por este último. El paciente fue tratado con 1 mg/kg de prednisona oral y 600 mg/día de valanciclovir durante 10 días, y terapia física. Quince días después del inicio del tratamiento, el paciente estaba completamente recuperado y no reportaba otros síntomas asociados.

## Discusión

Se reporta un caso de parálisis facial posterior a una infección reciente por CHIKV. Consideramos que el inicio de una parálisis facial diez días después de un episodio de infección por CHIKV confirmado clínica y serológicamente en un adulto previamente sano, respalda la posibilidad de postular al virus como agente etiológico. El CHIKV es un arbovirus del género *Alphavirus* de la familia Togaviridae, transmitido por la picadura de mosquitos hembra de la especie Aedes, principalmente *Ae. aegypti* y *Ae. albopictus*^4^. En Colombia, este virus fue epidémico en el 2015; entre el 2014 y el 2018 se reportaron 488.996 casos ^5^, y según la OMS, durante el 2017 se reportaron 1.052 casos ^1^.

Este virus se ha asociado con distintas manifestaciones neurológicas ^6^^-^^8^. En una revisión reciente de casos en Brasil, se reportó que, entre 1.410 pacientes atendidos en el Servicio de Neurología, el 14 % (n=201) presentaba síntomas indicativos de infección por arbovirus. La confirmación diagnóstica arrojó: 20 % con ZIKV, 27 % con CHIKV, 1 % con DENGV y 25 % con infección simultánea por ZIKV y CHIKV.

Estos pacientes presentaban una amplia gama de enfermedades del sistema nervioso central y del periférico. La infección por virus de chikunguña se asoció más frecuentemente con enfermedades del sistema nervioso central (47 % de los 55 pacientes con infección por CHIKV frente a 6 de 41 con infección por ZIKV; p=0,0008), especialmente, mielitis (12 pacientes).

Por otra parte, la infección por virus del Zika se asoció con mayor frecuencia de enfermedades del sistema nervioso periférico (26 de 41 pacientes con infección por ZIKV frente a 9 de 55 con infección por CHIKV; p≤0,0001), especialmente, síndrome de Guillain-Barré (25 pacientes). En los pacientes con este síndrome e infección simultánea por ZIKV y CHIKV, la enfermedad era más agresiva que en aquellos con monoinfección y que exigió el ingreso a cuidados intensivos. Por último, hasta 8 de los 46 pacientes con infección simultánea por ZIKV y CHIKV sufrieron un ictus o accidente isquémico cerebral transitorio, en comparación con 5 de 96 pacientes con monoinfección por ZIKV o CHIKV (p=0.047) ^7^.

En este y otros estudios, se resalta el amplio espectro de afecciones neurológicas inducidas por este virus: encefalitis, meningoencefalitis, mielopatía, síndrome de Guillain-Barré, hipotonía neonatal, enfermedad neuroocular y, menos frecuentemente, neuropatía craneal ^7^^,^^8^. Algunos reportes de casos aislados han asociado la infección por CHIKV con neuritis óptica y parálisis del tercer nervio craneal ^8^^-^^10^. Asimismo, el CHIKV se ha asociado con parálisis facial en el contexto de un síndrome de Guillain-Barré acompañado de otra enfermedad del sistema nervioso periférico ^8^^,^^11^^,^^12^. No obstante, el presente caso es el primero en que se describe el compromiso aislado del nervio facial periférico.

Aunque los estudios sobre la neuropatogenia de la infección por CHIKV son escasos, en algunos se ha demostrado su tropismo neurológico ^4^^,^^13^. Por ejemplo, se ha planteado que el CHIKV activa los astrocitos, lo cual conduce a hipertrofia de la sustancia blanca astrocitaria, lo que posteriormente alteraría el número y la distribución de las sinapsis neuronales. En otros estudios, se ha encontrado una relación entre el perfil de citocinas y las complicaciones neurológicas por el CHIKV, con concentraciones de TNF-a, IFN-a, IL-6 y monocina significativamente altas en pacientes con compromiso neurológico ^14^.

Por otra parte, en el contexto de la actual pandemia por Covid-19, se ha reportado que la infección por SARS-CoV-2 puede presentarse con neuropatías como la parálisis del nervio facial ^15^^,^^16^. En algunos reportes de casos, se ha descrito parálisis facial aislada y unilateral en pacientes positivos para SARS- CoV-2 ^15^ y, bilateral, en pacientes con síndrome de Guillain-Barré ^17^.

Se han formulado diversas hipótesis sobre los mecanismos por los que el SARS-CoV-2 puede desencadenar este tipo de neuropatías ^17^, planteándose que, de forma similar al CHIKV, activaría distintos mecanismos de daño inmunomediado por citocinas proinflamatorias ^17^. Asimismo, se ha descrito que los coronavirus presentan características neurotrópicas que pueden ocasionar daño nervioso directo ^18^^,^^19^, lo que también podría ser el caso de arbovirus como el CHIKV y el ZIKV.

En este contexto, hay fundamento para considerar que un paciente con parálisis facial puede tener una infección viral y que la Covid-19 debe sumarse al diagnóstico diferencial. En pacientes con parálisis periférica del nervio facial, se requiere un examen neurológico cuidadoso para descartar la afectación concomitante de otros nervios craneales (nervios trigémino y vestibulococlear) ^20^. En pacientes con parálisis del nervio facial, se debe considerar la probabilidad de una infección viral y no debe suponerse que sea idiopática, pues el tratamiento oportuno y adecuado podría mejorar el pronóstico.

Este reporte de caso aporta información adicional sobre los síntomas neurológicos asociados con el CHIKV y, hasta donde pudimos comprobar, es el primer caso con indicios de que este virus puede causar parálisis facial. Las muestras fueron analizadas en el Instituto Nacional de Salud de Colombia, ya que la prueba de IgG para este virus no se hace de forma rutinaria.

El diagnóstico diferencial de la parálisis facial es amplio y deben considerarse probables causas vasculares, neoplásicas, traumáticas e infecciosas de tipo viral. En un paciente con parálisis facial de aparición repentina y confirmación de una infección reciente por CHIKV, debe considerarse este virus como causa probable e incluirse en el diagnóstico diferencial de la parálisis facial. Además, en el presente caso, las pruebas para DENV y ZIKV fueron negativas, lo que excluye a estos otros arbovirus como causantes de la condición descrita. La enfermedad de Lyme no se consideró en el diagnóstico diferencial porque es poco frecuente en Colombia y el paciente no refirió ninguna exposición reciente a garrapatas. Por último, no se hicieron exámenes serológicos para virus del herpes simple (HSV), dada la ausencia de exantema facial y el resultado positivo de IgM para chikunguña, lo que indicaba una baja probabilidad de parálisis de Bell asociada con el virus del herpes. Además, el paciente negó haber tenido infección previa por este virus.

## Conclusión

El virus de chikunguña debe considerarse en el diagnóstico diferencial de la parálisis facial aislada concomitante con infecciones confirmadas en regiones endémicas o en pacientes con viajes recientes a estas regiones. En los casos de parálisis del nervio facial, debe considerarse comúnmente la probabilidad de una infección viral y no debe suponerse su origen idiopático, pues el tratamiento oportuno y adecuado podría mejorar su pronóstico.
